# Structural stability of the evolving developer collaboration network in the OSS community

**DOI:** 10.1371/journal.pone.0270922

**Published:** 2022-07-08

**Authors:** Liu Peng, Ma Jianan, Li Wenjun

**Affiliations:** School of Economics and Management, Jiangsu University of Science and Technology, Zhenjiang, China; Rutgers University, UNITED STATES

## Abstract

The structural stability of the developer collaboration network is critical to the success of the OSS (Open Source Software) community. However, research on the structural stability of the evolving developer collaboration network in OSS communities is relatively insufficient. In this paper, according to the software version sequence, we construct the corresponding developer collaboration network of the Angular OSS community and then analyse this network’s structural stability during network evolution. The results show that the network always presents an economical modular small-world structure during its evolution. The maintenance of the structure is related to a cohesive core, which is composed of two types of nodes (i.e., hubs and connectors). The hubs organize noncore nodes to form modules, while connectors facilitate the formation of inter-module connections. The overall results highlight the important role of core developers in the sustainable development of OSS communities and may provide a reference for community initiators to implement protection strategies for core developers.

## 1. Introduction

In recent years, open-source software (OSS) has flourished, and its application is widely used in many fields, such as artificial intelligence, virtual reality, and big data. According to the GitHub (a famous code hosting platform for OSS) 2020 report, the number of code repositories hosted by the platform is more than 200 million, up 43% compared to 2019, and the proportion of enterprise OSS has exceeded 50%. Undeniably, OSS has gradually become one of the major trends in the software industry [1].

Unlike commercial software, OSS is developed by the online community, where community members are worldwide volunteers who accomplish code writing and debugging through spontaneous collaboration [2, 3]. Correspondingly, the community members weave a network by engaging in collaborative relationships that act as pipes that facilitate the flow and integration of knowledge between community members [4].

In essence, the developer collaboration network of the OSS community is a self-organized complex network that forms as a result of the developers’ self-selected behaviours (e.g., freely joining and leaving the community, spontaneously finding a partner, etc.). Certainly, the structure of the developer collaboration network will affect the success of the OSS community. A fundamental theory in network science is that the function of real systems is reflected through the structural characteristics of their corresponding networks [5–8]. Empirical studies show that the developer collaboration network of successful OSS communities often has specific structural characteristics [9–14], such as modular [15] and small-world [4] structures. Meanwhile, there is evidence that an OSS community can fail during development, frequently due to insufficient participation by volunteers [16, 17]. From the perspective of complex networks, such insufficient participation manifests as structural changes in the developer collaboration network (e.g., network fragmentation because of the loss of nodes and links) during its evolution. Thus, maintaining structural stability during the evolution of developer collaboration networks is important to the success of OSS communities. The analysis of the structural stability of evolving developer collaboration networks is of great significance in understanding the sustainable development of OSS communities.

Many studies analysing the structural stability of networks have been conducted in different fields, such as transportation networks [18, 19], scientific collaboration networks [20, 21], and biological networks [22]. For example, Li et al. proposed a new metric (i.e., Markov criticality) to analyse the vulnerability of networks and verified its effectiveness in nine real-world networks [23]. The prosperity of OSS, such as Linux and Firefox, has also inspired studies on the structural stability of developer collaboration networks in OSS communities [24–26]. For example, Oh and Jeon used the Ising model to analyse the stability of developer collaboration networks in Linux and Hypermail communities and found that the scale-free characteristics of these two networks make them more stable than random networks. By analysing the developer collaboration network of the Python community, Sharma et al. found that a small number of developers act as boundary spanners who play an important role in the integration of the network. Through this brief review, it can be seen that the research on the structural stability of developer collaboration networks in OSS communities is relatively insufficient; in particular, the structural stability during the temporal development of such networks is worthy of further discussion.

**Fig 1:**
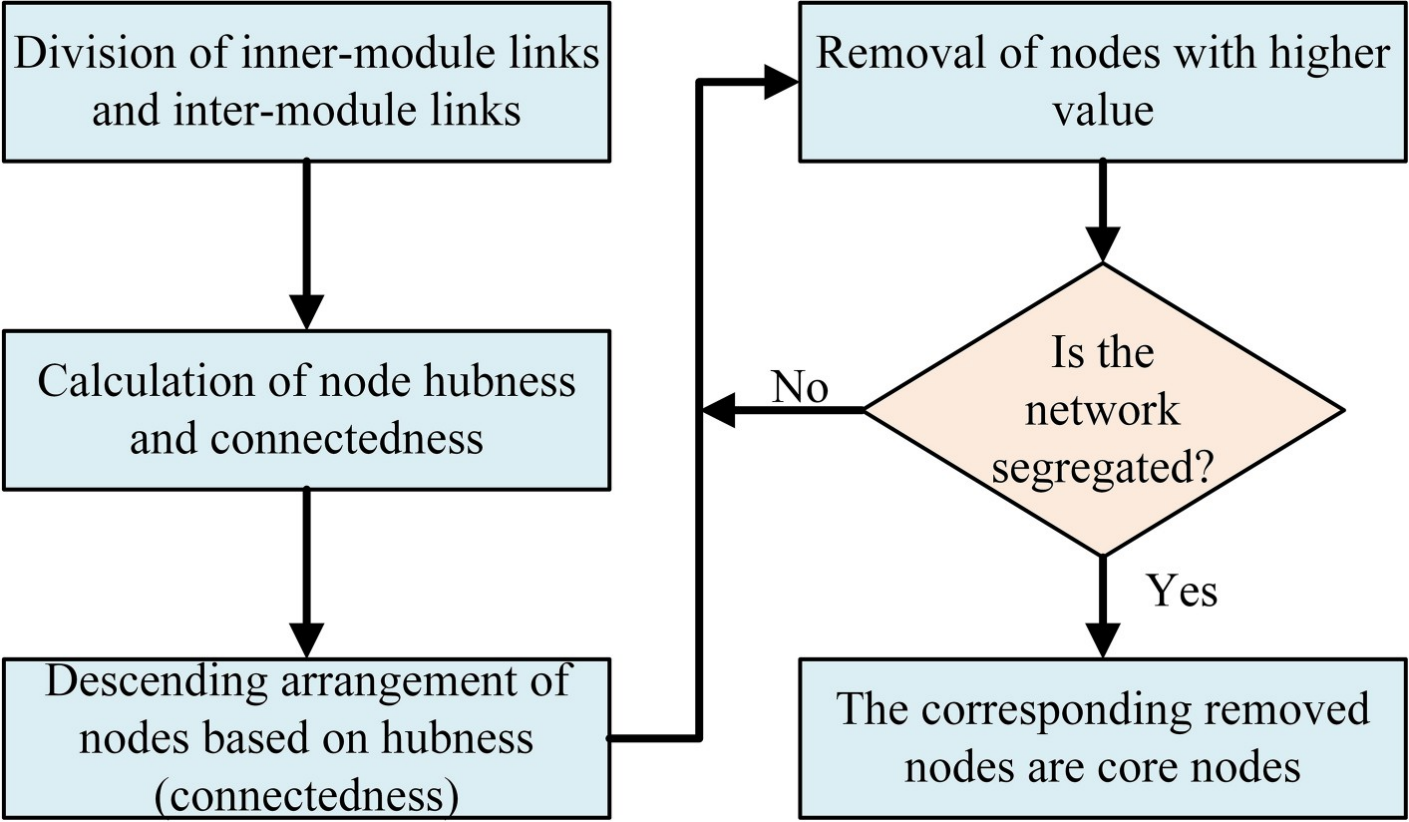
The flow chart of core node identification. Specifically, the links are divided into inner-module links and inter-module links. Then, for each module, the hubness (connectedness) of nodes is calculated according to the mean and standard deviation of their inner-module (inter-module) links. Nodes are sorted in descending order based on hubness and connectedness. Finally, nodes with higher values are continuously removed until the remaining network is segregated and the corresponding removed nodes are the core nodes.

Based on the above facts, the present study addresses a central research question: does the developer collaboration network of a successful OSS community always present some specific structures during its evolution, and if so, how are these specific structures maintained? Accordingly, in this paper, we investigate the structural stability of the evolving developer collaboration network of the Angular OSS community as a case in point. Among the network structures, our research focuses on modular and small-world structures, since these structures are two prominent properties of complex networks and affect the function of networks [6, 27]. For example, the modules (cluster communities) have essential scientific significance for understanding the clustering degree of individual interactions in real networks [28, 29]. Methodically, we analyse the structural maintenance of the network by identifying core nodes, which play an important role in the integration of network structure [30]. Our results show that, with the update of the software version, the collaboration network always presents a modular small-world structure, which not only inherently associates with the projects in software development but also achieves efficiency in the network through low wiring costs. The maintenance of such a structure is related to two types of core nodes (i.e., hubs and connectors), which are detected by hubness and connectedness, as shown in Fig 1. Generally, the contributions of the presented work are as follows.

1. We unveil the essence of the structural stability of the evolving developer collaboration network in OSS communities. In detail, the core nodes are relatively stable during network evolution and play the role of a cohesive core for the integration of network structure, where the hubs organize noncore nodes to form modules while connectors facilitate the formation of inter-module connections.
2. As opposed to existing studies that analyse the structural stability of networks only through node degree, we combine the node degree and the modular structure of networks and propose two metrics (i.e., hubness and connectedness) to capture the core nodes within and between modules. The empirical results of this approach indicate that these two metrics can effectively identify the core nodes that maintain network connectivity, especially the core nodes with nonhigh degrees. The combination of hubness and connectedness thus provides a new tool for the structural stability analysis of modular complex networks.

The remainder of this paper is organized as follows. Section 2 introduces the research dataset and the method of network construction. In Section 3, we examine the topological and economical properties of the network and then identify and analyse the core nodes that maintain the stability of the network structure. The work as a whole is summarized and discussed in Section 4.

## 2. Data and methods

### 2.1 Data and network construction

We investigate the structural stability of the evolving developer collaboration network in the Angular OSS community. Angular is an excellent open-source front-end framework at present. Its first version was developed in JavaScript in 2010, mainly for desktop applications. Since version 2, the community has rewritten version 1 in a different development language (i.e., TypeScript) to meet the requirement that the software support both PC and mobile applications, and these subsequent versions are incompatible with version 1. Thus, we collect the developer commitment information of the Angular community from version 2 to version 11 (May 2016 ~ November 2020) as the dataset and acquire 362080 commitment records in doing so. These commitment records cover nine major versions and six projects, and each commitment record contains the developer’s full name and email address, code changes and files involved, and the date-time of the commitment. It should be note that the developers did not use version 3 in the naming of major release versions to ensure the consistency of version numbers between the components and the software.

Using the acquired dataset, we construct the developer collaboration network for each major version. First, we collect the email addresses of each developer through the personal web page and conduct unified processing. Then, the developers are distinguished by email address and denoted as nodes. Second, each major version of Angular contains many patch releases, which are low risk and bug fixing releases; correspondingly, we consider two developers to have a collaborative relationship if they contributed code to the same file during the circle of a patch release, and the number of coding collaborations between two developers is expressed as the weight of the collaborative relationship. On this basis, we use the identified relationships to construct the developer collaboration network for each major version, and each network is named with the abbreviation of the major version number, such as the *v*2 and *v*4 networks, representing different versions of the network.

### 2.2 Methods

For the constructed network, we adopt several statistical metrics to investigate the structural properties of the network. The network size is measured by the number of nodes. The normalized metric *G* = *G*_0_/*G*_*n*_ is used to measure network connectivity, where *G*_0_ represents the size of the giant component and *G*_*n*_ represents the size of the entire network. The degree (*k*) represents the number of link-neighbours of each node. In addition to these basic metrics, we use the modularity (*Q*) obtained by the Louvain algorithm to examine the modular structure of the network. The relative clustering coefficient (*C* = *C*_*real*_/*C*_*rand*_) and the relative average shortest-path-length (*L* = *L*_*real*_/*L*_*rand*_) are used to examine the small-world structure of the network, where *C*_*real*_ and *L*_*real*_ represent the clustering coefficient and the average shortest-path-length of the real network, respectively, and *C*_*rand*_ and *L*_*rand*_ are the corresponding counterparts of the equivalent random network.

**Fig 2:**
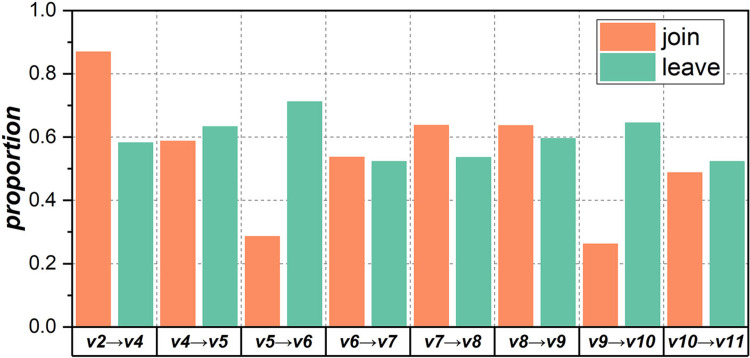
Variation among developers during the process of major version releases.

Fig 2 shows the variation among developers during the process of major version releases. Compared with the previous version, in each new version, the proportion of developers joining the community (i.e., the orange bars) ranges between 0.25 and 0.87, while the proportion of developers leaving the community (i.e., the green bars) ranges from 0.5 to 0.7. In addition, the proportion of developers joining and leaving the community is often significantly different. This result indicates that the variation among developers for each major version is significant and further reflects the necessity of studying the stability of developer collaboration networks in OSS communities.

## 3 Results

### 3.1 Topological and economical properties of the developer collaboration network

#### 3.1.1 Structural evolution of the network

Table 1 lists the basic properties of the developer collaboration network, including the number of nodes, average degree, and the relative size of the giant component. In Table 1, during network evolution, the network size corresponding to different software versions varies greatly, which indicates that there are obvious changes in community members. The average degree (*k*) is between 4.36 and 6.38, and the network always has a giant component with a large scale (*G* > 0.8). This result implies that large-scale collaboration has formed among community members and that the number of partners for each member is relatively stable.

**Table 1:** Basic properties of the developer collaboration network.

network	nodes	average *k*	*G*
*v2*	237	5.78	0.93
*v4*	301	5.19	0.94
*v5*	287	4.98	0.94
*v6*	187	4.72	0.83
*v7*	184	4.36	0.85
*v8*	197	6.38	0.88
*v9*	197	5.94	0.91
*v10*	123	5.64	0.90
*v11*	128	4.84	0.84

**Fig 3:**
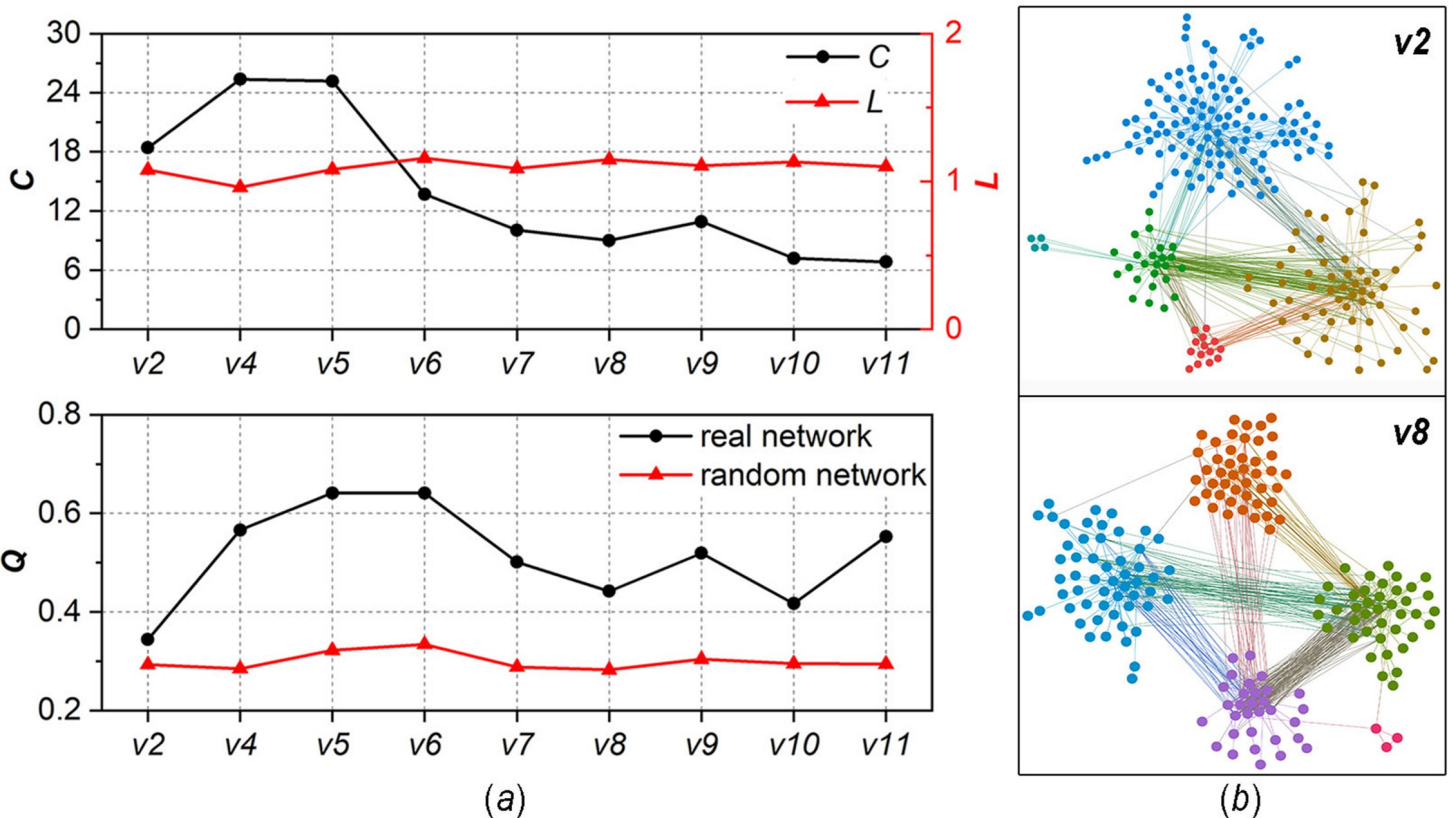
Characteristics of structural evolution of the giant component, (a) evolution of *C*, *L*, and *Q*, (b) topology of sample networks.

Fig 3 illustrates the characteristics of the structural evolution of the giant component. In Fig 3A, as the network evolves from *v2* to *v11*, the relative average shortest-path-length does not change significantly (*L* ≈ 1). Although both the relative clustering coefficient and modularity show fluctuating changes, their values are higher than those of the random network with the same scale (6 < C < 27, 0.3 < *Q* < 0.7). This result indicates that the giant component presents different degrees of modular small-world state during network evolution. Fig 3B exemplifies the topology of two networks, where the modules are distinguished by different colours. It can be observed that both networks show a clear modular structure; i.e., the network can be divided into some subnetworks, which have dense internal links and sparse external links. By further comparing the two networks, it can be seen that the density of inner-module links in the *v8* network is higher than that in the *v2* network, indicating that the modular structure of the *v8* network is more significant. The reason for this phenomenon may be that the *v2* version of software is only a rewrite of the *v1* version, while the *v8* version is an improvement of the previous version, and the increase in the corresponding workload leads to close collaboration between developers in the same module. As pointed out by Singh, such small-world characteristics are closely related to the success of OSS communities [4].

**Fig 4:**
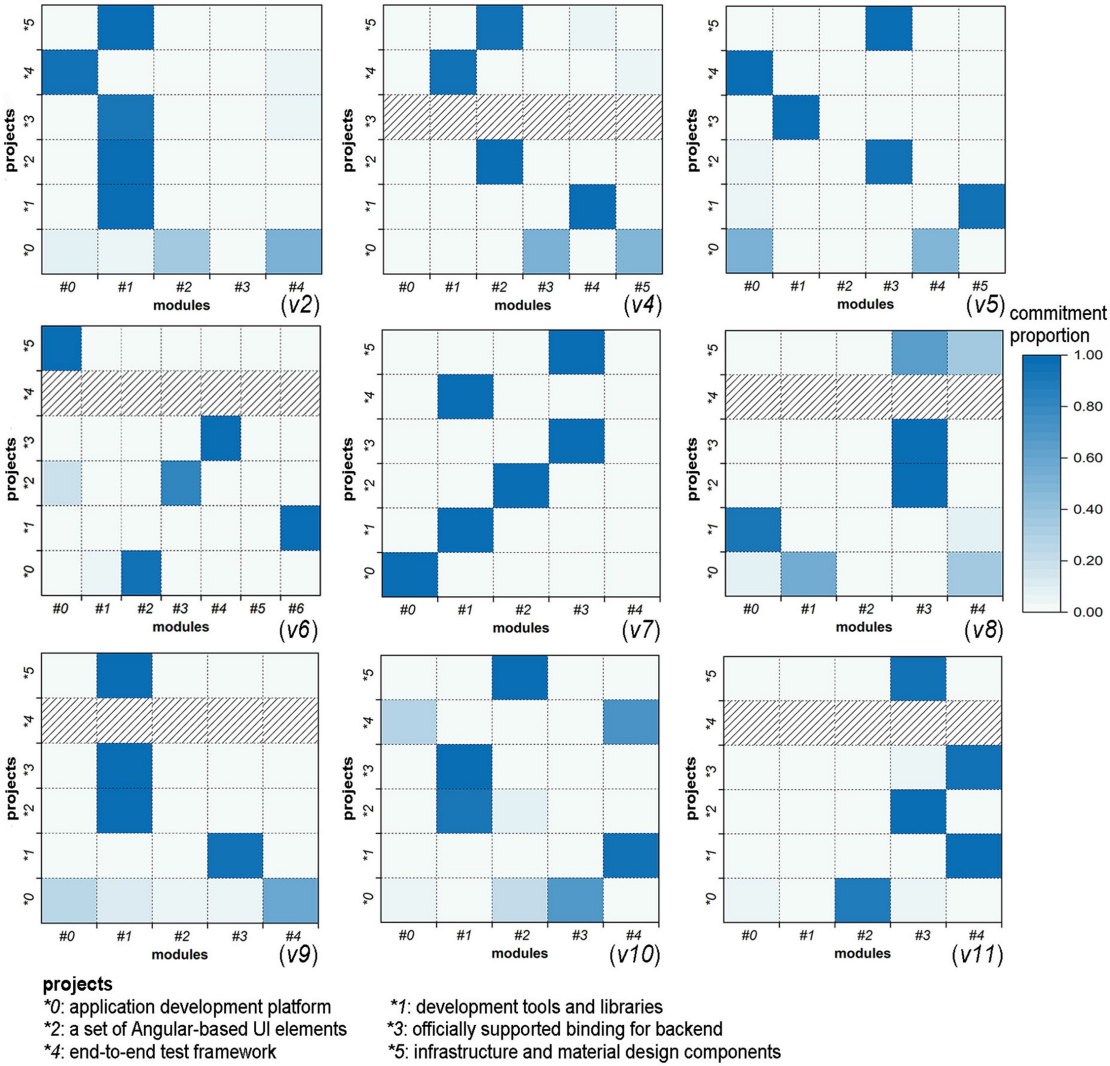
Relationship between modules and projects.

To analyse the relation between network modules and software projects, for each project, we counted the proportion of commitments contributed by modules, as shown in Fig 4. It can be seen that there is a division of labour among modules in the maintenance of the project. On the one hand, most of the work for each project is completed by a few (no more than three) modules. For example, in network *v5*, project *0 is maintained by modules #*0* and #*4*; projects *2 and *5 are maintained by module #*3*, and projects *1, *3, and *4 are maintained by modules #*5*, #*1*, and #*0*, respectively. On the other hand, although some modules maintain multiple projects, most modules only contribute code to a few projects. For example, in network *v*6, module #0 mainly participates in the development of two projects, while each of the remaining modules submits commitments to a specific project. The above results indicate that the projects are inherently related to the modular small-world structure of the network. In other words, the change in the modular structure (e.g., the separation of modules from the network) will inevitably affect the software development process. Thus, in the case of continuous changes of community members, the maintenance of this structure is particularly important for the orderly development activities of the community.

#### 3.1.2 Efficiency analysis of the giant component

We further test the efficiency of the modular small-world structure for the giant component of the examined network. Existing studies show that the small-world structure of different networks (e.g., neural networks, communication networks, and transportation networks) is efficient at the local and global levels [19, 31]. The efficiency between a pair of nodes is defined as the smallest sum of the physical distances throughout all the possible paths between them (i.e., the reciprocal of the shortest distance between the nodes). Correspondingly, the global efficiency of a network *G* (denoted as *E*_*g*_) can be obtained through Eq 1, where *n* represents the number of nodes and *L*_*ij*_ represents the shortest path length between nodes *i* and *j*. The local efficiency of a node is defined as the global efficiency of the subnetwork composed of the neighbours of such a node, and the local efficiency of the network *G* (denoted as *E*_*l*_) is the average of all nodes’ local efficiencies.


$$\eta =\frac{1}{n(n-1)}\sum_{i\neq j}\frac{1}{L_{ij}}$$ (1)

**Fig 5:**
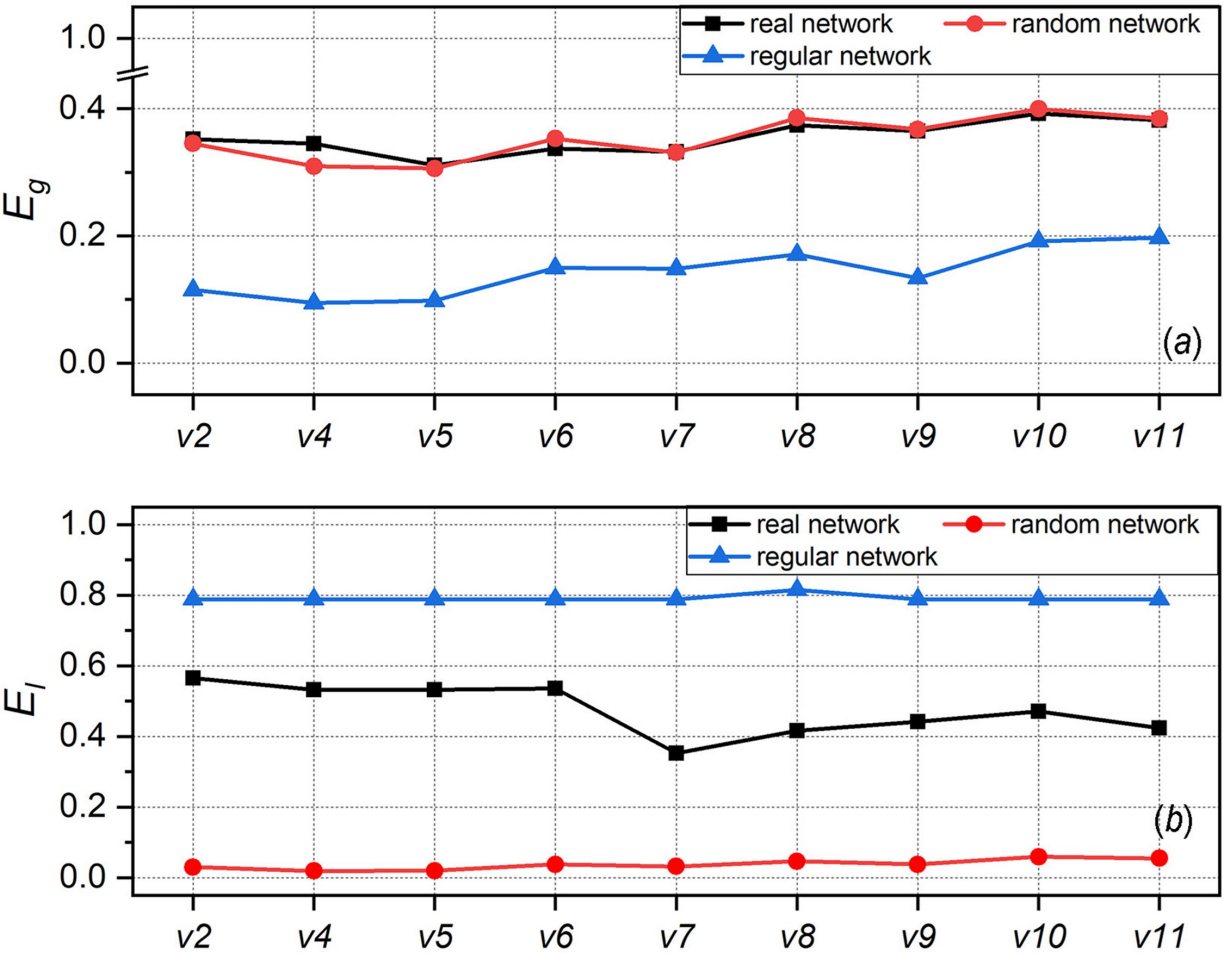
Efficiency of the developer collaboration network.

Fig 5 illustrates the efficiency of the examined network and compares it with that of the equivalent random network and regular network. Theoretically, the random network has the highest global efficiency because the distance between nodes is small, while the regular network has the highest local efficiency because of tight connections among local nodes. For the global efficiency (*E*_*g*_), as shown in Fig 5A, the examined network (the black line) is close to the random network (the red line) and much higher than the regular network (the blue line). In Fig 5B, the local efficiency of the examined network (*E*_*l*_) is lower than that of the regular network but higher than that of the random network. This implies that the modular small-world structure of the examined network achieves a trade-off between global efficiency and local efficiency. Furthermore, the links between nodes can be regarded as the cost of network formation; thus, regardless of the structure of the regular network or of the random network, it is necessary to increase the number of links (i.e., pay more cost) to achieve such a trade-off. The examined network is also an economical small-world network.

### 3.2 The role of core nodes in network structural stability

#### 3.2.1 Identification and analysis of core nodes

This section analyses the reasons for the existence of structural stability during network evolution by identifying core nodes. For the identification of core nodes, two metrics are used, namely, hubness and connectedness. The first metric, hubness, measures how well-connected a node is to other nodes in its module and is obtained by Eq 2. *z*_*i*_ is the hubness of node *i*, *k*_*i*,*in*_ is the sum of its weighted links to other nodes in its module *m*, and *k*_*in*,*m*_ and *S*_*in*,*m*_ are the average and standard deviation of *k*_*i*,*in*_ in *m*, respectively. The second metric, connectedness, reflects the participation degree of a node in the inter-module links of its module, as shown in Eq 3. *p*_*i*_ is the connectedness of node *i*, *k*_*i*,*out*_ is the sum of inter-module weighted links of module m that node *i* has participated in, and *k*_*out*,*m*_ and *S*_*out*,*m*_ are the average and standard deviation of *k*_*i*,*out*_ in *m*, respectively.


$$z_{i}=\frac{k_{i,in}-{\bar{k}}_{in,m}}{s_{in,m}}$$ (2)

$$p_{i}=\frac{k_{i,out}-{\bar{k}}_{out,m}}{s_{out,m}}$$ (3)

**Fig 6:**
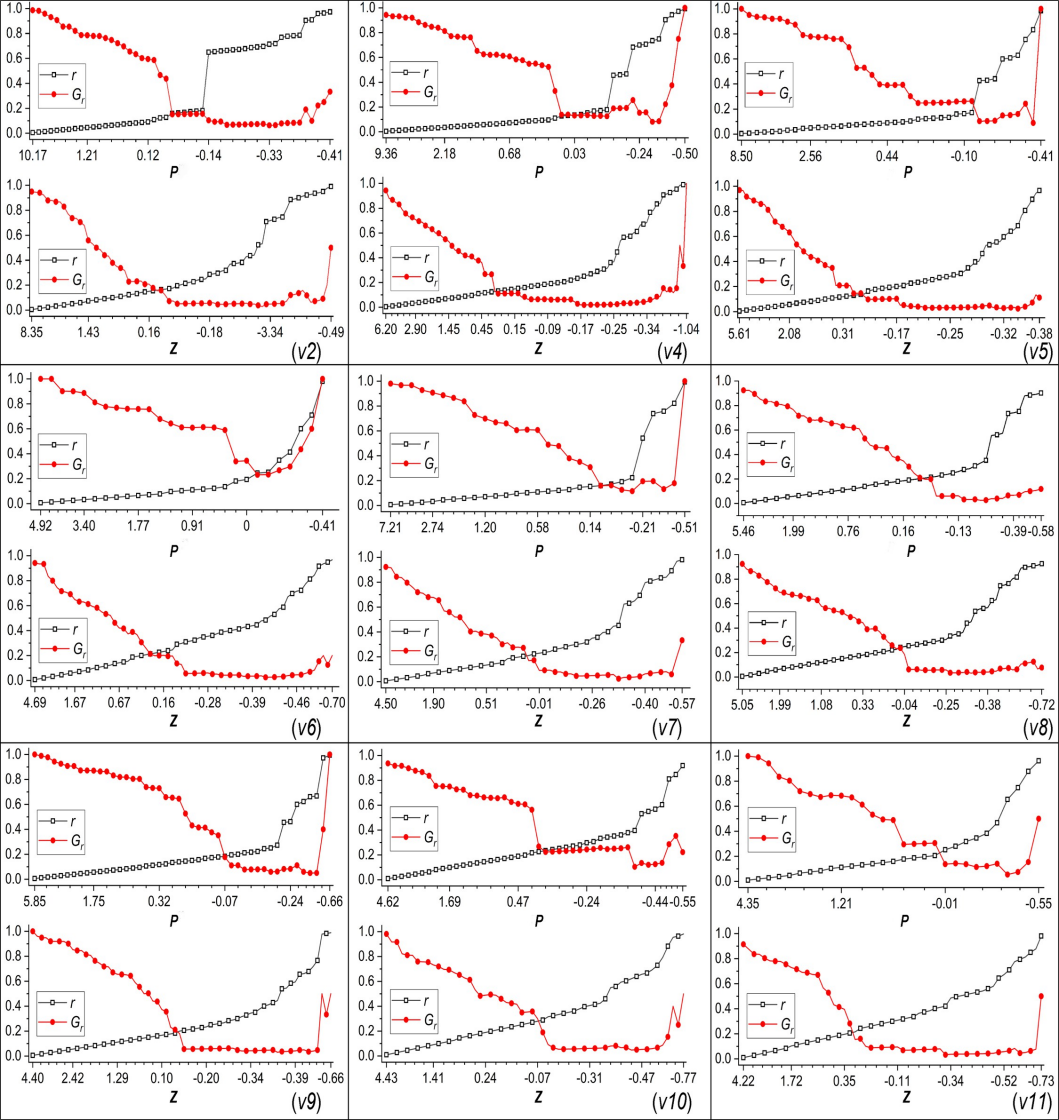
Changes in network connectivity during node removal processes.

Based on the hubness and connectedness of nodes, we explore the role of nodes in the maintenance of network structures by removing them. First, the nodes are arranged in descending order according to their hubness (connectedness). Then, the nodes with higher values are continuously removed, and the connectivity of the remaining network is calculated (i.e., the relative size of the giant component of the remaining network, denoted as *G*_*r*_, is calculated). Fig 6 shows changes in the network connectivity after removing nodes, where *r* represents the proportion of removed nodes. With the decrease in *p* (*z*), the proportion of removed nodes gradually increases, while the connectivity of the remaining network continuously decreases and then increases only when the remaining network size is very small. For illustration, we describe the network *v*5 as representative of the primary result. In the removal process based on *p*-value, with the removal of *p* > 1.0 nodes, the connectivity of the remaining network gradually decreases to approximately 0.7. After further removing *p* > 0.44 nodes, the connectivity of the remaining network rapidly decreases (*G*_*r*_ < 0.4), and the proportion of removed nodes is approximately 0.1. In the subsequent removal process, the connectivity decreases slowly and recovers until the proportion of removed nodes reaches 0.9. In the removal process based on *z*-value, with the gradual removal of *z* > 0.31 nodes, the connectivity of the remaining network decreases sharply to below 0.3, and when the nodes with higher *z* are further removed, the decline in connectivity slows down. These results indicate that some nodes in the network play the roles of module hub and connector, which are further identified in Table 2.

**Table 2:** Identification of hubs and connectors.

giant component	core nodes					
	connectors		hubs		proportion (%)	code contribution (%)
	*p*_*c*_	proportion (%)	*z*_*c*_	proportion (%)		
*v2*	-0.03	12.7	0.33	11.3	15.5	75.7
*v4*	0.15	10.9	0.45	11.3	15.2	90.4
*v5*	0.05	10.4	0.41	10.7	14.8	90.4
*v6*	0.00	19.4	0.32	20.0	29.0	88.3
*v7*	0.14	15.3	0.16	18.5	22.9	88.9
*v8*	0.14	19.1	0.15	20.8	24.9	89.2
*v9*	-0.05	17.8	0.03	17.2	21.1	93.3
*v10*	0.37	21.6	-0.07	27.9	31.5	92.7
*v11*	0.51	15.9	0.35	19.6	23.4	80.6

As shown in Table 2, we regard the remaining network with *G*_*r*_ < 0.3 as a segregation network in the node removal process and take the corresponding *p* and *z* values as thresholds (denoted as *p*_*c*_ and *z*_*c*_, respectively) for identifying the hub (connector) when *G*_*r*_ is lower than 0.3 for the first time. Correspondingly, the core nodes are composed of *p* > *p*_*c*_ nodes (hubs) and *z* > *z*_*c*_ nodes (connectors). It can be seen in Table 2 that although the *p*_*c*_ and *z*_*c*_ values vary greatly, the respective proportions of hubs and connectors in the giant component are not more than 30%, and the proportion of core nodes is less than the sum of the proportions of these nodes, indicating that some nodes are both hubs and connectors. In addition, the core nodes have contributed more than 75% of the code for the community. This implies that a small number of nodes (i.e., the core nodes) not only have an important impact on the maintenance of network structures but also carry considerable weight in code contribution.

**Fig 7:**
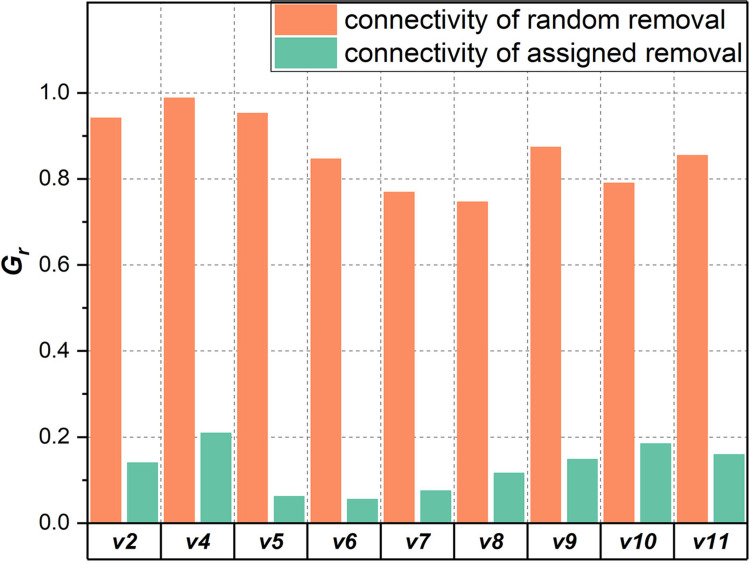
Comparison of the connectivity of the remaining network under random and assigned removals.

Fig 7 further detects the maintenance effect of the core node on the network structure, where the green bar represents the connectivity of the remaining network after removing core nodes (i.e., assigned removal) and the orange bar represents that of the remaining network after removing the same proportion of randomly chosen nodes (i.e., random removal). The connectivity of random removal is the average of the results of fifty rounds of repeated random removal. Under the assigned removal, the connectivity of the remaining network shrunk to a very low level (*G*_*r*_ < 0.3). Combined with the proportion of removed nodes (as shown in Table 2), we can affirm that the remaining network represents a segregation state. In comparison, under random removal, the *G*_*r*_ value remains above 0.7, which indicates that the giant component of the remaining network contains most nodes; accordingly, randomly removing a small number of nodes will not cause fatal damage to the connectivity under such a removal regime.

**Fig 8:**
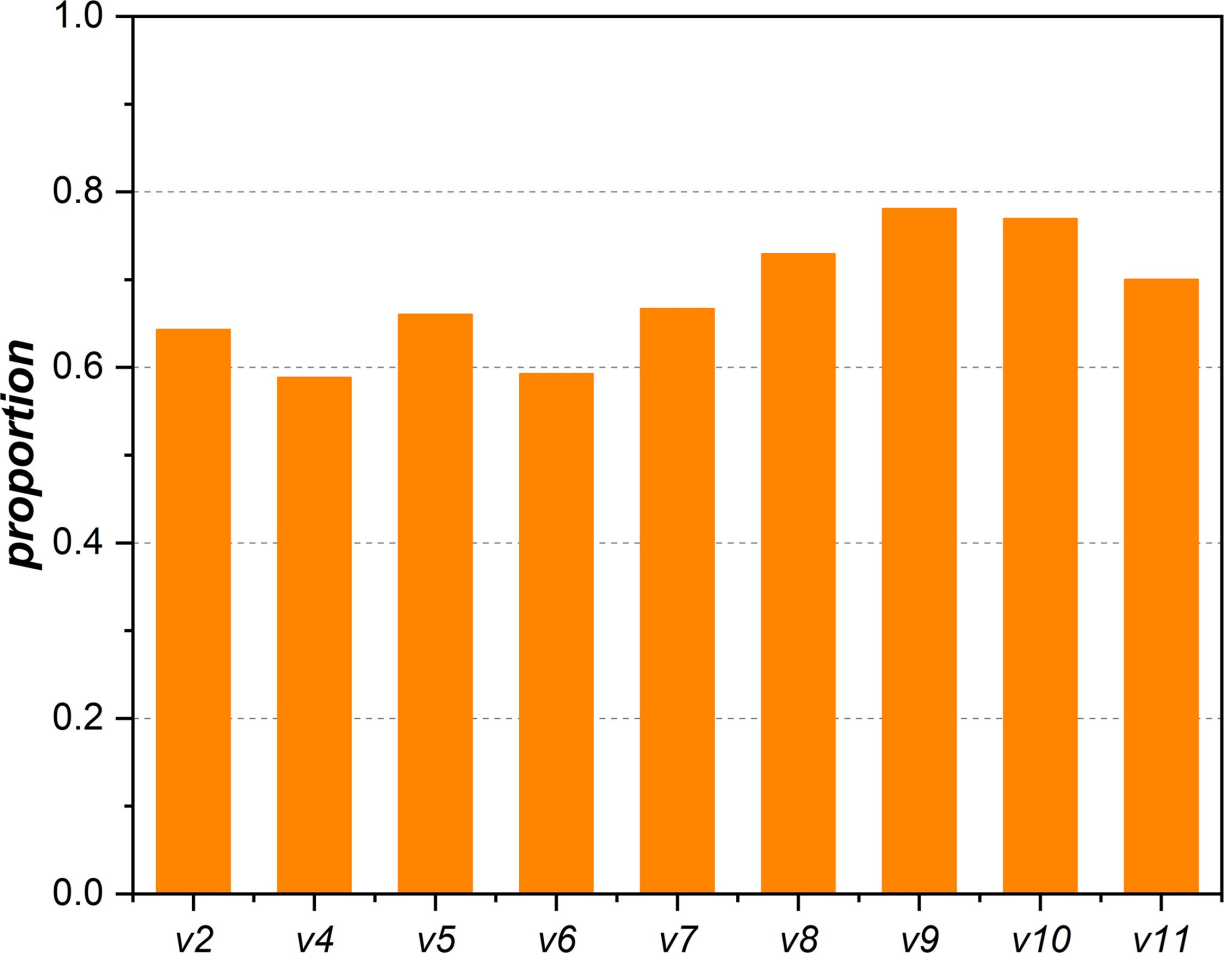
Overlap of core nodes and high-degree nodes.

To examine whether the core nodes are composed of high-degree nodes, we extract the proportion of high-degree nodes from a given network that matches the proportion of core nodes present in that same network and analyse the overlap between them, as shown in Fig 8. It can be observed that the proportion of overlap between core nodes and high-degree nodes is not more than 0.65 when the network evolves from *v*2 to *v*7. Starting with the network *v*8, the proportion increases and stays between 0.7 and 0.8. Although many core nodes are high-degree nodes, 20%~40% of core nodes with nonhigh degrees also play a crucial role in structural stability during network evolution. This indicates that, in addition to the core nodes with a high degree, the combination of hubness and connectedness can also effectively identify core nodes with a nonhigh degree.

**Fig 9:**
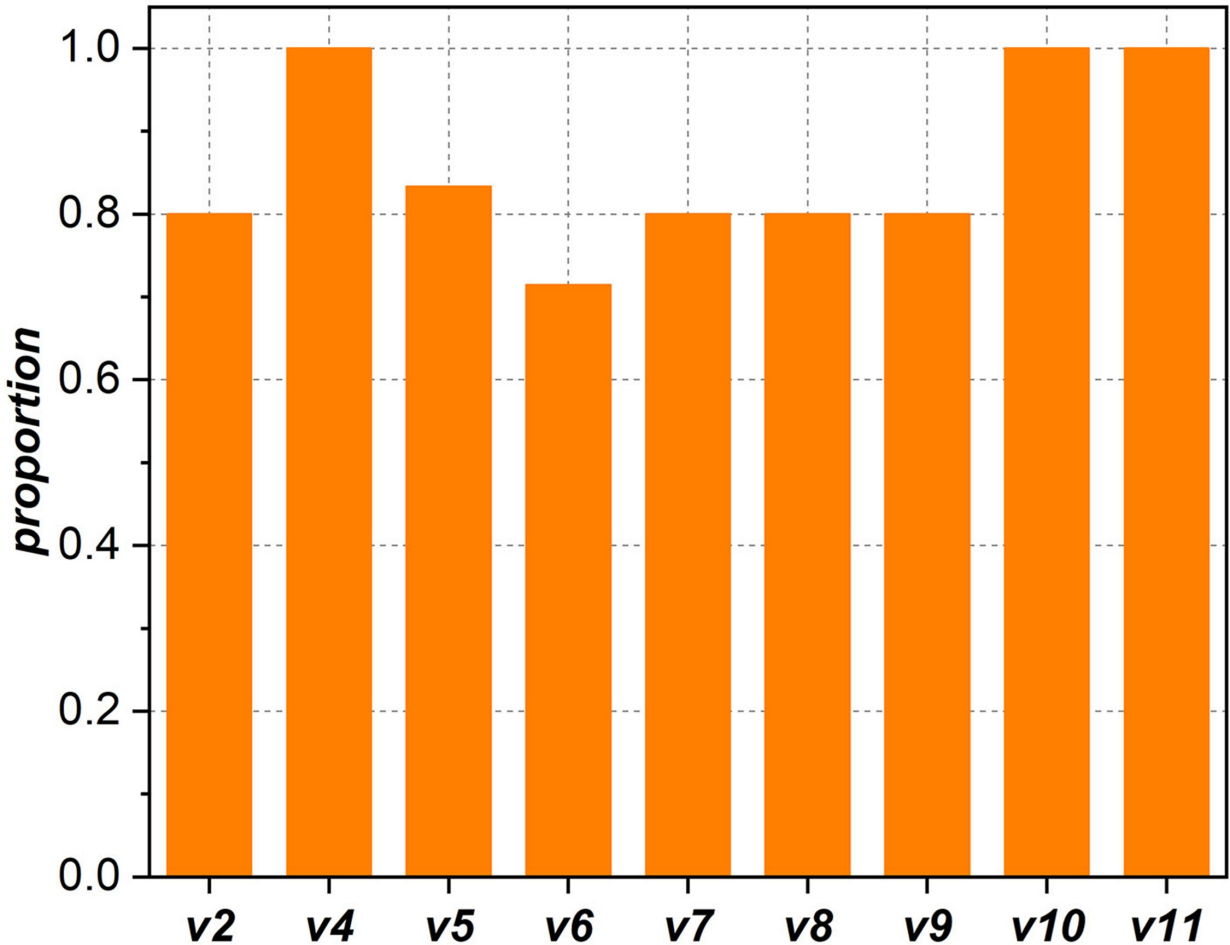
Proportion of modules containing both a hub and a connector.

Fig 9 shows the distribution of core nodes in modules. During network evolution, the proportion of modules with a hub and a connector in the giant component is between 0.7 and 1.0, demonstrating that the core nodes are scattered in most modules. This also further reflects the internal structure of the giant component: the modules are often formed around a few hubs and connected with each other through the collaboration provided by connectors.

**Fig 10:**
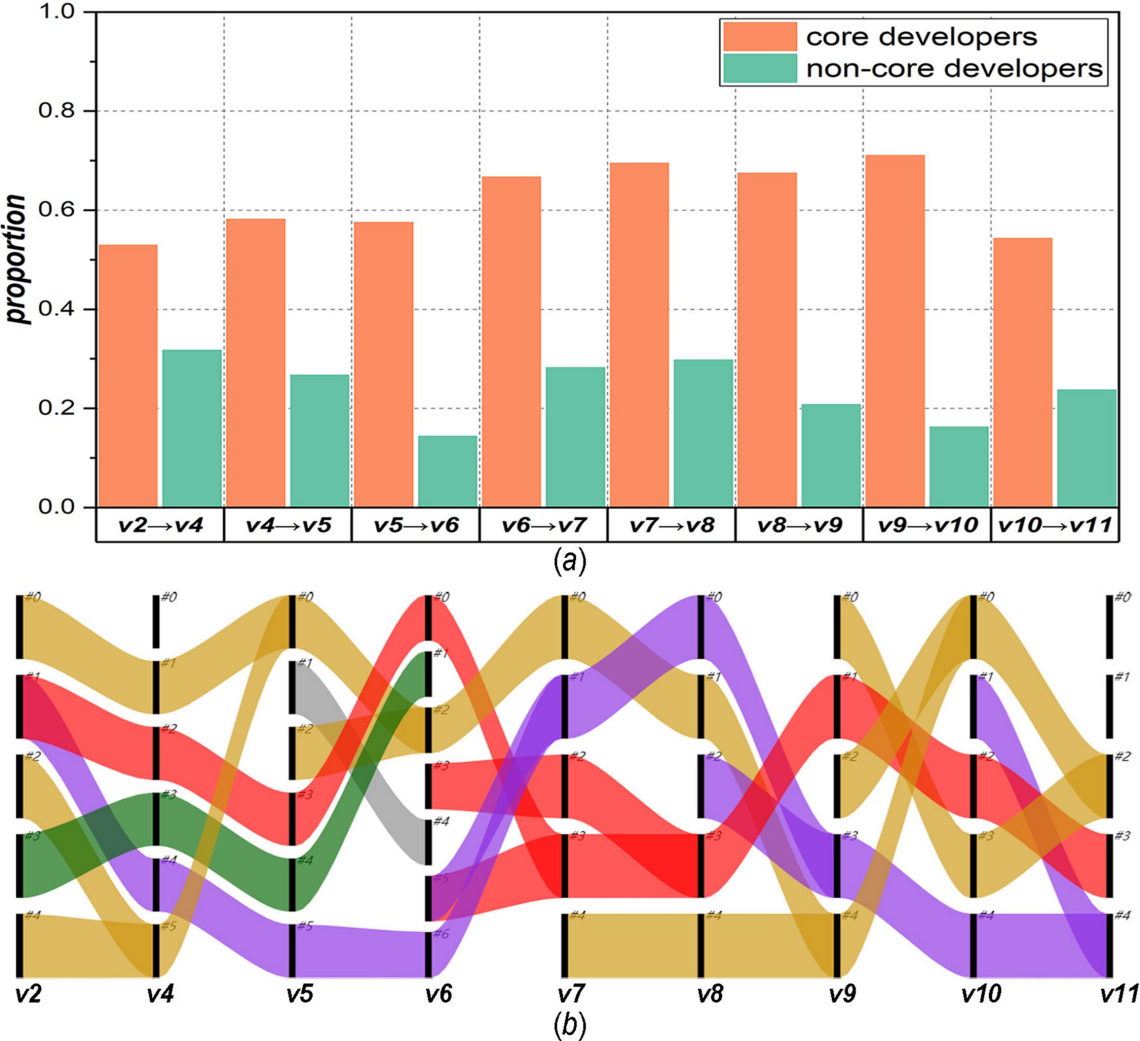
Changes in core nodes during network evolution, (a) retention proportion of core and noncore nodes, (b) evolution of core nodes.

We also test the changes in core nodes (developers) during network evolution, as shown in Fig 10. Compared with the network corresponding to the previous software version, Fig 10A illustrates the retention proportion of two kinds of nodes (i.e., core and noncore nodes) in the network corresponding to the current software version. When the network evolves from *v*2 to *v*11, the retention proportion of core nodes is more than 0.5, and the highest value can reach approximately 0.7, while that of noncore nodes is basically no more than 0.3. This demonstrates that in the process of network evolution, the core nodes are relatively stable, but the noncore nodes change greatly. Fig 10B further shows the flow of core nodes during network evolution, where the short black line represents the module, and the coloured flow between short lines represents the flow direction of core nodes. We can observe that in the process of network evolution, although some core nodes no longer play their role, new core nodes emerge and maintain the network structure together with the retained core nodes. For example, in network *v*6, the core nodes of modules #3 and #5 are newcomers, and those of modules #0, #2, and #6 are retained nodes, while the core nodes of modules #1 and #4 no longer act as core nodes in network *v*7.

#### 3.2.2 Cohesive core composed of core nodes

The previous result exhibits the role of core nodes in maintaining the modular small-world structures of the collaboration network. However, we are not clear about the connection between these core nodes, especially about whether they collaborate closely together. This is an interesting question worthy of further examination. To test this, we use the “clubness coefficient” (denoted as *θ*) to measure the connection between nodes. The clubness coefficient is the ratio of the number of actual links to the number of potential links for the examined nodes, as shown in Eq 3, where *e* is the number of actual links among n nodes. Then, starting from the subgraph comprised of core nodes, we restore the network by continuously adding noncore nodes and calculate the clubness coefficient of each network after adding nodes.


$$\theta =\frac{e}{n\left(n-1\right)/2}$$ (4)

**Fig 11:**
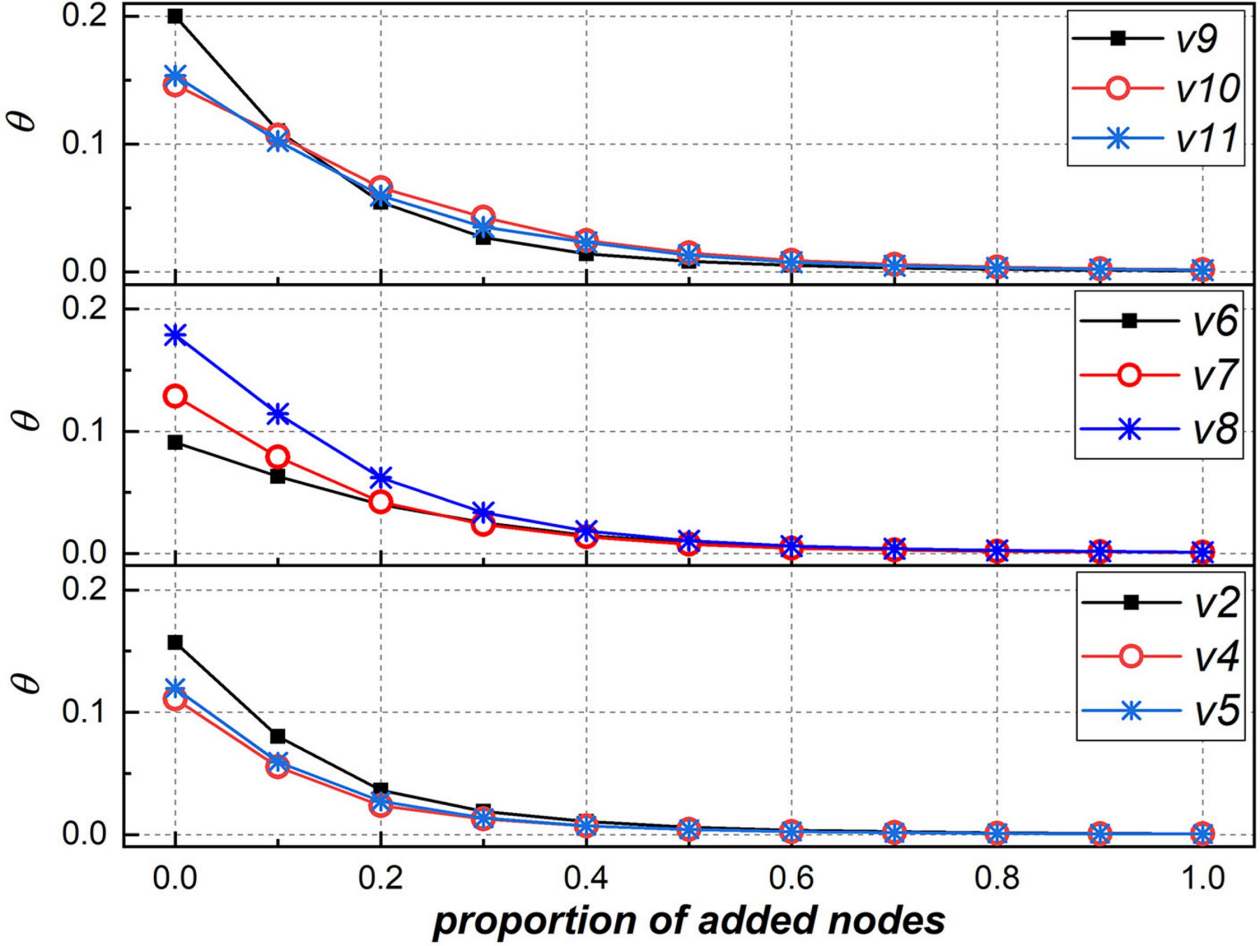
Variations in the clubness coefficient of the giant component.

Fig 11 shows the variations in the clubness coefficient of the giant component during the node addition process. For each giant component, when the subgraph is only composed of core nodes (i.e., the proportion of added nodes is zero), the clubness coefficient is the highest. With the proportion of added nodes gradually increasing to 0.2, the clubness coefficient decreases sharply (*θ* < 0.05), and the decrease in the clubness coefficient slows down with the further addition of noncore nodes (*θ* → 0). This result indicates that core nodes are more closely connected and that the connections between noncore nodes are relatively sparse. From this, we can assert that core nodes form a cohesive core of the network, which plays a skeleton role in organizing noncore nodes to form a modular small-world structure.

**Fig 12:**
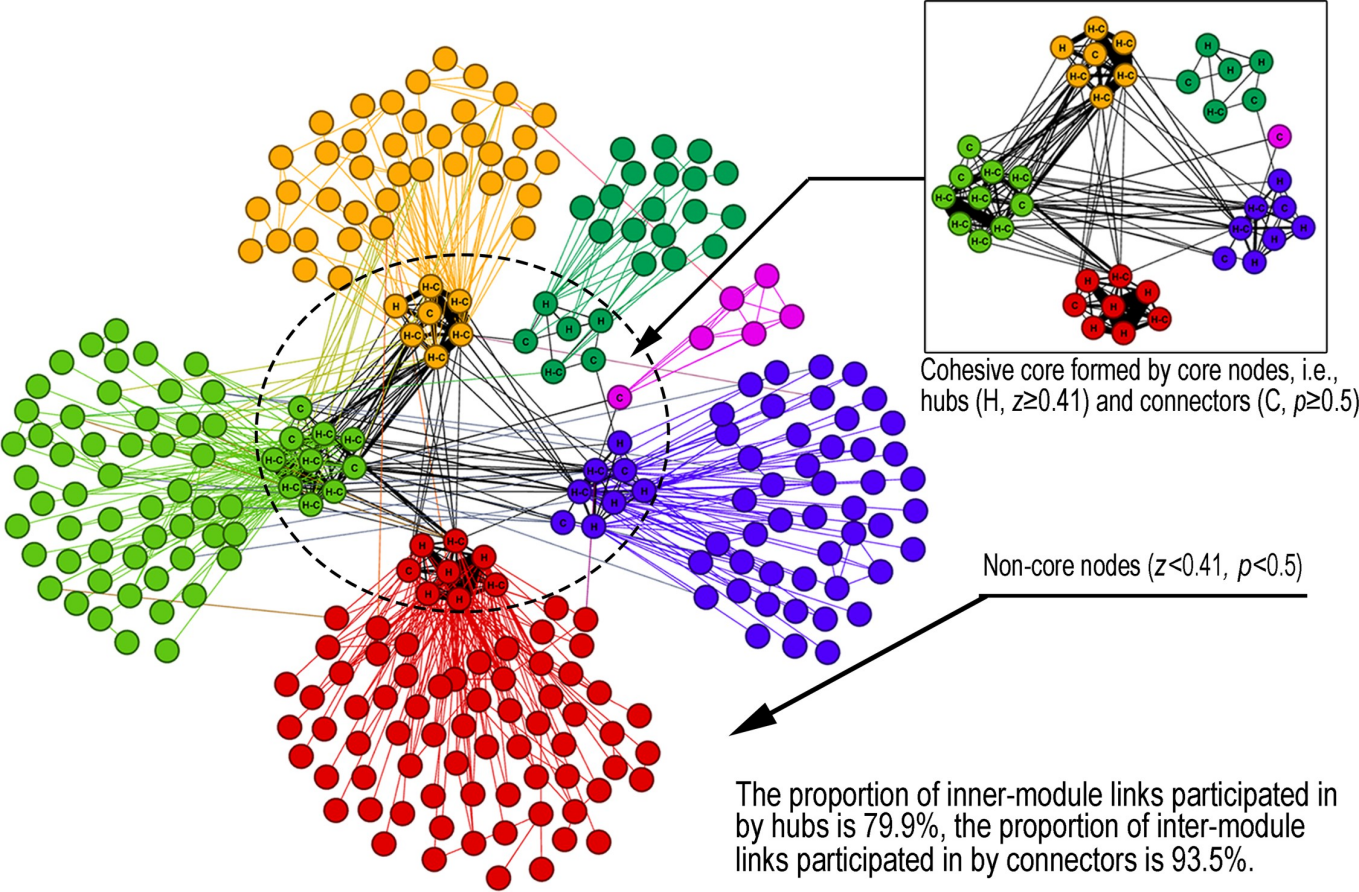
Maintenance role of core nodes in network structure integration.

Taking network *v*5 as a representative example, Fig 12 summarizes the maintenance role of core nodes (i.e., hubs and connectors) in network structure integration. Each module contains a small number of core nodes (the nodes in the dashed circle), and these core nodes are tightly connected to form the cohesive core of the network (as shown in the subgraph). Correspondingly, the modular small-world structure of the network is formed centred on such a core. More than half of the inner-module links are participated in by the hubs (the nodes marked with “H”), indicating the module organizing role of hubs. The connectors (the nodes marked with “C”) participate in most inter-module links, which is key to maintaining the integrity of the network. In addition, some core nodes are both hubs and connectors (the nodes marked with “H-C”), which play a dual role in structural integration.

## 4. Conclusion

This paper analyses the structural stability of the developer collaboration network in the Angular OSS community. Our findings show that the network presents a modular, cohesive and small-world structure during its evolution, and the maintenance of such a structure is closely related to the properties of a small number of nodes. The main findings of this paper are summarized as follows.

First, with the update of the software version, although the members of the corresponding network change greatly, the network always presents a modular small-world structure, which is inherently related to the projects involved in software development. In addition, compared with the random network and the regular network, the modular small-world structure of the examined network achieves a trade-off between global efficiency and local efficiency through lower wiring costs. Therefore, the examined network is also an economical small-world network.

Second, the structural stability of the examined network during its evolution is achieved by a small number of stable core nodes, which are composed of hubs and connectors. Specifically, these core nodes appear in most modules and are closely connected to form the cohesive core of the network, which plays a key role in the integration of the network structure. The hubs integrate noncore nodes to form modules, while the connectors promote the formation of inter-module connections.

Finally, the two metrics (i.e., hubness and connectedness) proposed in this paper combine node degree and modular structure to realize the subdivision of nodes, which can effectively identify the core nodes that maintain network connectivity, especially for modular networks. In addition, an empirical result discovered in this study (as previously described in Fig 8) shows that some nonhigh degree nodes identified by the two metrics also play a crucial role in the maintenance of network structures.

However, there are some limitations in the present study. First, although we have obtained some interesting results by examining the structural stability of the developer collaboration network in the Angular community, the present work, which is limited to the analysis of a single case, is still far from a solid theory for explaining the social dynamics of developer collaborative relationships and the development of OSS communities in general. Second, with respect to the analysis method used in this paper, the notion of “connectedness” that we use mainly focuses on the number of inter-module links of nodes, without considering the distribution of such inter-modular links among all modules. The different distributions of nodes’ inter-module links often may have different effects on the maintenance of network connectivity. In the future, to extend the examination of the developer collaboration network offered in the present work, we will examine the multilayer network that combines the developer collaboration network with its corresponding communication network and knowledge network. We expect that such an extensive study may provide a comprehensive understanding of the sustainable development of OSS communities. In addition, the proposed method will be further extended by incorporating the examination of the distribution of nodes’ inter-module links to enable a more detailed exploration of the role(s) that core nodes play during network evolution.

## 5. Code ability

The code for community detection is available at https://igraph.org/python/api/latest/igraph._igraph.GraphBase.html#clusters. All other codes used in this study are available from the corresponding author upon reasonable request.

## Supporting information

S1 Data (ZIP)
